# Multi-Feature Classification of Multi-Sensor Satellite Imagery Based on Dual-Polarimetric Sentinel-1A, Landsat-8 OLI, and Hyperion Images for Urban Land-Cover Classification

**DOI:** 10.3390/s18020373

**Published:** 2018-01-27

**Authors:** Tao Zhou, Zhaofu Li, Jianjun Pan

**Affiliations:** College of Resources and Environmental Sciences, Nanjing Agricultural University, Nanjing 210095, Jiangsu, China; xinyangzhoutao@163.com (T.Z.); lizhaofu@njau.edu.cn (Z.L.)

**Keywords:** Sentinel-1A, random forest, urban area mapping, Hyperion, Landsat-8, multi-sensor, multi-feature

## Abstract

This paper focuses on evaluating the ability and contribution of using backscatter intensity, texture, coherence, and color features extracted from Sentinel-1A data for urban land cover classification and comparing different multi-sensor land cover mapping methods to improve classification accuracy. Both Landsat-8 OLI and Hyperion images were also acquired, in combination with Sentinel-1A data, to explore the potential of different multi-sensor urban land cover mapping methods to improve classification accuracy. The classification was performed using a random forest (RF) method. The results showed that the optimal window size of the combination of all texture features was 9 × 9, and the optimal window size was different for each individual texture feature. For the four different feature types, the texture features contributed the most to the classification, followed by the coherence and backscatter intensity features; and the color features had the least impact on the urban land cover classification. Satisfactory classification results can be obtained using only the combination of texture and coherence features, with an overall accuracy up to 91.55% and a kappa coefficient up to 0.8935, respectively. Among all combinations of Sentinel-1A-derived features, the combination of the four features had the best classification result. Multi-sensor urban land cover mapping obtained higher classification accuracy. The combination of Sentinel-1A and Hyperion data achieved higher classification accuracy compared to the combination of Sentinel-1A and Landsat-8 OLI images, with an overall accuracy of up to 99.12% and a kappa coefficient up to 0.9889. When Sentinel-1A data was added to Hyperion images, the overall accuracy and kappa coefficient were increased by 4.01% and 0.0519, respectively.

## 1. Introduction

Over the past few decades, the world has undergone an unprecedented process of urbanization and it is estimated that by 2030, 60% of the global population will live in cities [1]. The rapid population growth has caused unhealthy housing, air pollution, traffic congestion, food security and other issues in urban areas. With the acceleration of urbanization, many changes have taken place in the spatial layout and functions of cities, and the regional ecosystems and climate have been affected [2]. Accurate and timely collection of reliable urban land use and land cover (LULC) information is the key to addressing these issues and achieving sustainable urban development, which is also important for urban planners and decision-makers. Due to its characteristics of frequent and large area detection, remote sensing technology has become an important means to obtain the information of LULC quickly, and has made great contributions to monitoring the process of dynamic urbanization. Extensive researches have been carried out to classify urban land cover using optical remote sensing data [3,4,5,6]. However, optical remote sensing images may not be available during key monitoring periods due to their vulnerability to cloud and rainy weather. Synthetic aperture radar (SAR) has become an effective data source for urban land cover mapping because of its ability to capture data independent of cloud cover or solar illumination conditions and its unique information content [7]. 

Many studies of urban land cover mapping based on SAR data have obtained promising classification results. Earlier studies mainly used single-frequency and single-polarization data for urban land cover identification. However, the limited information obtained from single-frequency and single-polarization SAR data leads to limited classification accuracy [8,9,10,11]. To improve the classification accuracy, many urban land cover identification studies have been conducted based on multi-temporal, multi-polarization and multi-frequency SAR data. Park et al. [12] used multi-temporal/polarization C-band SAR data for land-cover classification and found that multi-polarization SAR data can improve classification accuracy. Dell’Acqua et al. [13] used ERS-1/2 images for urban land cover identification and found that multi-temporal and multi-angle SAR data can produce better classification results. Ban et al. [14] conducted urban land cover mapping using RADARSAT Fine-Beam C-HH SAR data and QuickBird images, and found that their decision level fusion improved classification accuracy. Similar results were also reported by Waske et al. [15], Mazher et al. [16], and Bigdeli et al. [17], who used SAR and optical sensors to classify LULC by decision fusion. Some recent studies have demonstrated the usefulness of polarimetric decomposition information for urban land cover classification [18,19,20,21,22]. The multi-temporal capabilities of Sentinel-1 have the potential to improve the classification accuracy of LULC [23]. Accurate LULC mapping can be obtained using multi-temporal SAR images alone [24]. McNairn et al. [25] used a single date ASAR (VV, VH) image for LULC identification and found that the classification accuracy was less than 80%. Another study showed that four Sentinel-1A images were required to achieve a classification accuracy of better than 85% [26]. Therefore, multi-temporal Sentinel-1A data is very useful for LULC identification. The date selection of remote sensing images is also important. Deng et al. [27] examined the impact of seasonality on urban impervious surface mapping under different climate conditions and found that humid continental areas may prefer summer imagery, while the tropical monsoon and Mediterranean areas seem to favor autumn and winter images. 

In addition, different morphological characteristics of urban areas can be obtained from SAR and optical images [28]. First, many researchers have combined SAR with multispectral data for urban land-cover mapping. Werner et al. [29] combined RADARSAT-2 and SPOT-5 data for urban land cover classification and found that optical-radar image fusion improved classification accuracy. Shao et al. [30] combined the GF-1 multispectral and Sentinel-1A data for urban impervious surface extraction at decision level, with an overall accuracy of 95.33%. Jiang et al. [31] used the combination of optical and InSAR data for urban impervious surface mapping. Leinenkugel et al. [32] combined TerraSAR-X and SPOT-5 data for settlement detection and impervious surface estimation. Zhou et al. [33] used GF-1 PMS and Radarsat-2 data to estimate building densities and found that the combination of optical and SAR data has the potential to improve building density estimation performance. Yang et al. [34] combined the SPOT 5 and ERS-2 images to quantify the sub-pixel urban impervious surface and found that there is potential for monitoring urban settings with InSAR data. In addition to multispectral data, hyperspectral images can identify targets based on unique spectral signatures [35], so some researchers used the combination of SAR and hyperspectral images for LULC identification. Borghys et al. [36] used the combination of SAR and hyperspectral sensors to classify urban scenes and found that their fusion can significantly improve the classification accuracy. Shokrollahi et al. [37] compared the classification results based on the feature-level and decision-level fusion of Polarimetric SAR (PolSAR) and hyperspectral images. While much progress has been made in using SAR alone and its combination with optical data, urban land-cover mapping remains a challenge. For example, despite the good progress made in the two multi-sensor land-cover mapping methods described above, there are few comparative studies between them. The choice of classifiers is very important for the identification of LULC and many studies have demonstrated the effectiveness of random forest (RF) [38] in SAR data classification [39,40,41,42]. 

Furthermore, many features can be extracted from SAR images, and the most commonly used is the backscatter coefficient. For example, Skakun et al. [43] used multi-temporal C-Band Radarsat-2 intensity and Landsat-8 for crop identification. Shu et al. [44] extracted the shoreline from RADARSAT-2 intensity images. Li et al. [45] used a marked point process to detect oil spills from SAR intensity images. Since SAR data can provide abundant texture information, many previous studies have extracted texture features from SAR data for LULC information extraction. Some previous studies used the combination of texture and backscatter intensity features to map urban land cover and found that texture features were important information that can improve classification accuracy [46,47]. In addition to backscatter intensity and texture features, some researchers also extracted coherence and color features from SAR data for urban land cover classification. Uhlmann et al. [48] performed LULC classification by extracting color features from SAR data and demonstrated the usefulness of color features. Pulvirenti et al. [49] analyzed the role of coherence features in mapping floods in agricultural and urban environments. Xiang et al. [50] conducted urban mapping using model-based decomposition and polarization coherence. Zhang et al. [51] only quantitatively evaluated the contribution of texture features. Another study evaluated only the contribution of texture features obtained using three texture measures families [52]. Wurm et al. [53] performed slum mapping using multi-temporal X-band SAR data and evaluated the contribution of texture features by using RF. Schlund et al. [54] evaluated the contribution of texture and coherence features to forest mapping. Although the above studies obtained good classification results using different SAR-derived features, they only used part of these features and did not compare the contribution of these different feature types. Consequently, there are still only a few studies that make use of multiple feature types extracted from SAR data for urban land cover mapping, especially studies that quantitatively assess the contribution of different feature types. 

The objective of this study was to evaluate the ability and contribution of using backscatter intensity, texture, coherence and color features extracted from Sentinel-1A data for urban land cover classification and to compare different multi-sensor land cover mapping methods to improve classification accuracy. For this purpose, the backscatter intensity, texture, coherence, and color features were extracted from Sentinel-1A data and then the RF classifier was used to explore the contribution and potential of different combinations of these features to urban mapping. We then used the different combinations of Sentinel-1A, Landsat-8 OLI, and Hyperion images to compare and explore the complementary advantages of the three different optical and radar sensors. 

## 2. Study Area and Datasets

### 2.1. Study Area

**Figure 1:**
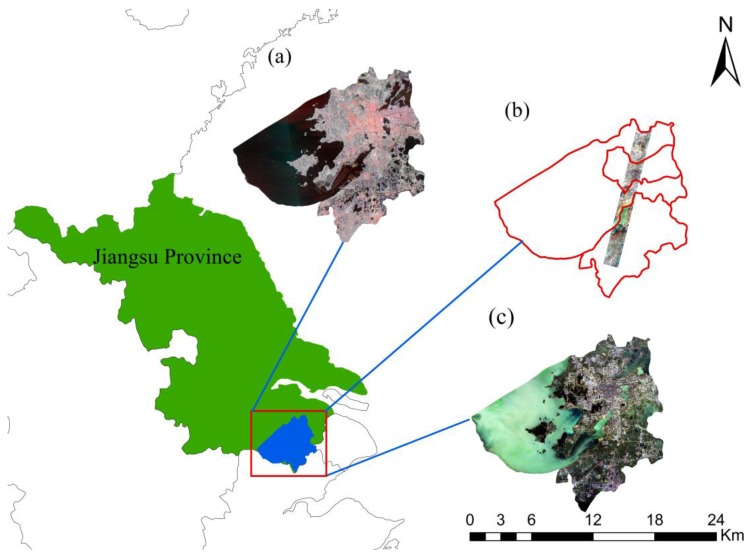
The research area is located in the Yangtze River Delta in eastern China; and an overview of the Sentinel-1A, Landsat-8 OLI, and Hyperion images: (**a**) The Sentinel-1A composite image (R: 18 August 2015 VV polarization, G: 11 September 2015 VH polarization, B: 25 July 2015 VH polarization), (**b**) The Hyperion composite image (R: band 29, G: band 20, B: band 12), (**c**) The Landsat-8 OLI composite image (13 October 2015, RGB = 432).

The study area is located in the downtown area of Suzhou (30°47′ to 32°02′ N; 119°55′ to 121°20′ E), Jiangsu Province, China (Figure 1). As one of the largest cities in the Yangtze River Delta, Suzhou has a total area of 8657.32 km^2^ and the permanent population was 10.616 million at the end of 2015. It has a subtropical monsoon climate with annual average temperature of 15–17 °C and annual average precipitation at around 1076 mm. The average elevation of Suzhou is about 4 m above sea level, plain area accounts for 54% of the total area; many hills in the southwest. In recent years, Suzhou has become a typical rapid urbanization area due to the rapid economic development and dramatic changes in land use. However, with the development of society and the improvement of economic level, the rapid urbanization has caused the rapid increase of urban population, environmental deterioration, and resource crisis and so on. In order to effectively address these issues, there is an urgent demand to monitor the dynamic urbanization in the area. 

### 2.2. SAR Satellite Data

As the first new space part of the GMES (Global Monitoring for Environment and Security) satellite series, Sentinel-1 is a constellation of two satellites designed and developed by ESA (European Space Agency) and funded by the European Commission. Sentinel-1A and Sentinel-1B were launched on 3 April 2014 and 25 April 2016, respectively [55]. With the launch of Sentinel-1B, double the amount of data was obtained and global coverage was achieved in six days. Sentinel-1A carries a C-band SAR instrument (with a 12-day repeat cycle [56]) and has the following four operational modes: Stripmap mode (SM), Interferometric Wide Swath mode (IW), Extra Wide Swath mode (EW), and Wave mode (WV) [57]. In this study, four Sentinel-1A images were obtained from ESA and detailed image information is shown in Table 1.

### 2.3. Optical Satellite Data

As a joint project of NASA and the U.S. Geological Survey (USGS), Landsat 8 was launched in February 2013 and has two sensors: Operational Land Imager (OLI) and Thermal Infrared Sensor (TIRS) [26]. The OLI sensor has nine spectral bands (bands 1–9), eight channels of 30 m spatial resolution, and one panchromatic channel with 15 m spatial resolution; the TIRS has two spectral bands (bands 10–11) [58]. A Landsat 8 OLI image on 13 October 2015 was obtained from the USGS with a cloud cover of 3.2%.

Hyperion is a Hyperspectral instrument on the Earth Observation 1 (EO-1) spacecraft launched on 21 November 2000 [59], with 7.5 km coverage [60]. It has a total of 242 bands from 357 to 2577 nm with a spectral resolution of 10 nm and a spatial resolution of 30 m [61,62]. In this study, one Hyperion image (ID: EO1H1190382015042110PF) of 11 February 2015 was captured from the USGS. In addition, a GaoFen-2 (GF-2) on 28 December 2015 was obtained and used as reference data. GF-2 was successfully launched on 19 August 2014. It is China’s first civilian optical remote sensing satellite with a spatial resolution better than 1 m. The GF-2 satellite carries two high-resolution 0.8-m panchromatic and 3.2-m multispectral cameras [63].

### 2.4. Accuracy Assessment 

In this study, six different urban land cover types were identified: dark impervious surface (DIS), bright impervious surface (BIS), forest (FOR), water (WAT) and grass (GRA). Impervious surface means any material that prevents water from penetrating the soil, which not only represents urbanization but also is a major contributor to the environmental impact of urbanization [64]. Impervious surfaces can be divided into two types according to their physical composition [65]: BIS (e.g., asphalt and old concrete) and DIS (e.g., new concrete and metal) [66]. A set of samples was obtained by visual interpretation of GF-2 image (image date: 28 December 2015) and with reference to Google Earth images with very high spatial resolution (image date: 8 December 2015). Then, using stratified random sampling, about 50% of the samples were used as the training samples and the remaining 50% were used to test the results and calculate the accuracy (Table 2). 

Finally, the confusion matrix was used to calculate the overall accuracy (OA) and kappa coefficient to evaluate the classification results of urban land cover. In addition, as the harmonic mean of the producer’s and user’s accuracy [67,68], the F1 measure (Equation (1)) was also calculated to evaluate the effectiveness of the urban land cover classification. The F1 measure is considered to be more meaningful than the kappa coefficient and the overall accuracy [69], and its values range from 0 to 1; a larger F1 measure indicates better results, and a smaller F1 measure indicates poorer results:

(1) $$F1\;\mathrm{measure}=2\times \frac{\mathrm{producer}\prime s\;\mathrm{accuracy}\times \mathrm{user}\prime s\;\mathrm{accuracy}}{\mathrm{user}\prime s\;\mathrm{accuracy}+\mathrm{producer}\prime s\;\mathrm{accuracy}}$$

## 3. Methods

### 3.1. Satellite Data Pre-Processing 

The preprocessing of Sentinel-1A data was conducted using SARscape 5.2 software, including the following process: multi-look, registration, speckle filtering, geocoding, and radiometric calibration. To reduce speckle, a Lee filter with 3 × 3 windows was applied to all Sentinel-1A images [70]. The digital number (DN) values of Sentinel-1A images were converted to backscatter coefficient (σ^0^) in decibel (dB) scale. The Sentinel-1A images were then geocoded using the shuttle radar topography mission DEM with a spatial resolution of 30 m. 

Preprocessing of the Landsat 8 OLI and GF-2 images using ENVI 5.3 included radiance calibration, atmospheric correction, and geometric correction. The DN value of the raw image was converted to the surface spectral reflectance by radiance calibration. The atmospheric correction of these multispectral data was conducted using the ENVI FLAASH model. The bad bands and bad columns of the Hyperion image were removed because many of their bands show low signal-to-noise ratio or other problems [59,71]. Atmospheric correction was then also implemented using the FLAASH algorithm. All images were also geometrically rectified using 25 ground control points (GCP), with the root mean square error (RMSE) less than 0.5 pixels.

### 3.2. Feature Sets 

#### 3.2.1. Color Features

Color features are important visual features, which is used to describe the visual content of the whole image or specific image area. Although they do not provide the natural color information of a target, they can provide useful information for understanding and analyzing Sentinel-1A data. Compared with grayscale images, color images not only have better visual display, but also have rich information about image details [72]. In order to explore more information to improve the classification accuracy of urban land cover, the HSV color space [73] was used to extract the color features of Sentinel-1A images from false color images. The HSV color space decomposes the color into hue (*Hu*), saturation (*Sa*) and value (*Va*) components [74], and the *Hu*, *Sa*, and *Va* values are the color features [75]. For the dual-polarized Sentinel-1A data used in this study, the two different polarization backscattering matrices were used to obtain pseudo color images (R: |VH|, G: |VH-VV|, B: |VV| or R: |VV|, G: |VV-VH|, B: |VH|). In this study, we assigned the |VH|, |VH-VV| and |VV| scatter matrices to red, green and blue image components, or assigned the |VV|, |VV-VH| and |VH| scatter matrices to red, green and blue image components. 

#### 3.2.2. Texture Features

Texture is an intrinsic spatial feature of an image [76] and is an effective representation of spatial relationship [77]. It is important for the application of SAR images because of the rich texture information in SAR images. Therefore, for remote sensing data, especially SAR data, texture is considered as an important tool to distinguish land cover types. In this study, the gray level co-occurrence matrix (GLCM) was used to extract the following texture features: mean, variance, correlation, dissimilarity, contrast, entropy, angular second moment, and homogeneity. Since the extraction of texture features is affected by the size of the selected window, texture measures at different window sizes were calculated to obtain the appropriate window size. To account for the different spatial variability patterns of the land cover types in the study area, empirical criteria were used to select the window size [78]. After several experiments, some different window sizes were tested: 3 × 3, 5 × 5, 7 × 7, 9 × 9, and 11 × 11 … 75 × 75. In order to reduce the influence of the direction and improve the extraction accuracy of the texture features [76], they are detected in four directions of 0°, 45°, 90° and 135°; finally, the final texture information was calculated from the average value of four directions.

#### 3.2.3. Coherence Features

Coherence is an estimate of the phase consistency of the imaged targets during the time interval between two SAR acquisitions [79]. The value of the coherence coefficient is between 0 and 1, and the coherence size determines whether the corresponding pixel has changed; a larger coherence coefficient indicates a smaller change, while a smaller coherence coefficient indicates a larger change. In this study, SARscape 5.2 software was used to calculate the coherence of Sentinel-1A data for both VV and VH polarizations and six coherence images were obtained using the following InSAR image pairs: (1) 1 July 2015 and 25 July 2015; (2) 25 July 2015 and 18 August 2015; and (3) 18 August 2015 and 11 September 2015. 

#### 3.2.4. Feature Combination

In order to assess the ability and contribution of using backscatter intensity, texture, coherence and color features extracted from Sentinel-1A data for urban land cover classification and to compare different multi-sensor land cover mapping methods to improve classification accuracy, the following combinations were considered (Table 3). The surface reflectance values in the spectral bands from Landsat-8 OLI and Hyperion data formed a feature vector that was input to the classifier. The images were then stacked in different combinations.

### 3.3. Classifiers 

Random forest is a machine learning method proposed by Breiman [38]. It is a classifier based on decision tree, in which each tree contributes one vote [78], and the final classification or prediction results are obtained by voting [80]. A large number of studies have shown that RF produces relatively high classification accuracy in SAR data classification [23,81,82]. According to [83], RF outperforms standard classification methods, such as a simple decision tree, because it allows for greater differentiation between different land cover classes; RF is relatively robust to training dataset reduction and noise; due to the Law of Large Numbers, RF does not overfit. For each RF classifier, a default value of 500 trees were grown using the square root of the total number of features at each node. In addition, it can handle large data sets [53] and also provides useful information about variable importance. In the present study, the variable importance was used to reduce the number of input variables. 

RF uses bootstrap aggregating to enhance the diversity of classification trees [78], and the samples that are excluded from the bootstrap sample are referred to as out-of-bag (OOB) samples [51]. The RF classifier assesses the importance of variables using the Gini index and the OOB subset [84]. According to [84], for a training data *T*, the Gini index can be defined as (Equation (2)):

(2) $$\sum \sum_{j\neq i}\left(f\left(C_{i},T\right)/\left|T\right|\right)\left(f\left(C_{j},T\right)/\left|T\right|\right)$$

where $f\left(C_{i},T\right)/\left|T\right|$ is the probability that the selected case belongs to class $C_{i}$.

## 4. Results and Discussion 

### 4.1. Texture Analysis of Sentinel-1A Image 

**Figure 2:**
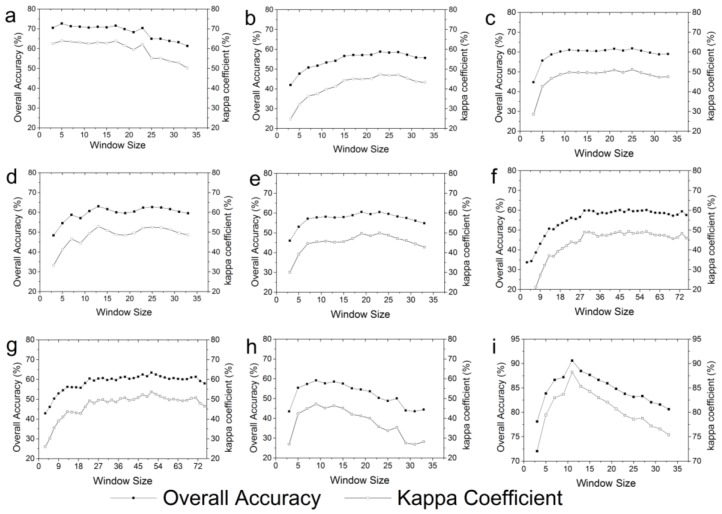
(**a**–**h**) are the classification results of each texture feature in different windows. (The corresponding sequences are mean, variance, entropy, dissimilarity, homogeneity, correlation, contrast and angular second moment). (**i**) is the classification result of the combination of all the texture features in different windows.

Figure 2 shows the classification accuracy of the texture feature experiments based on different window sizes in order to obtain the best identification window size. The combination of all texture features obtained the highest classification accuracy; in terms of window size, the best classification result was achieved with 9 × 9 (overall accuracy and kappa coefficient were about 90% and 0.88, respectively). 

According to the work of Pesaresi [85], image spatial resolution and land cover characteristics were the main determinants of the optimal window size for texture features. Wurm et al. [53] used the TerraSAR-X data to calculate the texture features of different window sizes for LULC identification, and found that the window size of 81 × 81 was the best. The classification accuracy using a single texture feature was lower than that of the combination of all the texture features. The classification results for each individual feature reveal the following: the mean feature had the best classification result, followed by the dissimilarity; the worst classification result came from the angular second moment and correlation features. The best classification results for each individual feature (mean, variance, entropy, dissimilarity, homogeneity, correlation, contrast and angular second moment) correspond to the following window sizes: 5 × 5, 23 × 23, 25 × 25, 13 × 13, 19 × 19, 49 × 49, 51 × 51 and 9 × 9, respectively, with overall accuracy of 72.70%, 58.81%, 61.82%, 63.08%, 60.55%, 60.31%, 63.39% and 59.13%. Before the best classification result was achieved, the classification accuracy increased with the increase of the window size, and then decreased with the increase of the window size after reaching the best classification result. This shows that the best classification results for different texture features correspond to different window sizes. A more effective representation of the most heterogeneous environments with high local variance can be obtained with the smallest window size. In contrast, a more accurate representation of the often homogeneous pattern of spatial variability of large areas can be obtained with the larger window sizes [78]. Compared with the previous studies [53,86], this paper not only explored the optimal window size of the combination of all the texture features, but also obtained the optimal window size corresponding to each individual texture feature. We chose the optimal window size corresponding to each individual texture feature for the following experiment.

### 4.2. Feature Selection

In this paper, all of the input features were 102 Sentinel-1A-derived features (i.e., color features, texture features, coherence features, and backscatter intensity features) and the importance of variables was assessed using RF classifiers (see Section 3.3).

**Figure 3:**
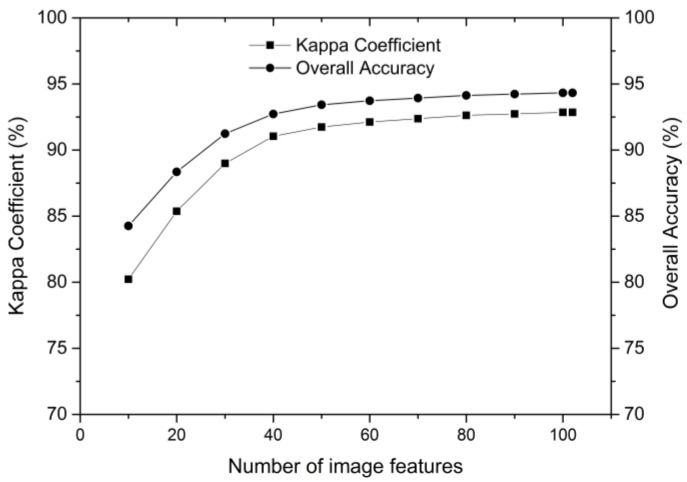
Effect of number of best features on classification accuracy using Sentinel-1A-derived features.

Figure 3 shows that the classification accuracy varies with the number of *n* best features selected. The overall accuracy and kappa coefficient obtained using the first 10 most important features were about 85% and 0.8000, respectively. As more features were added, higher classification accuracy was achieved. This improvement continued until 50 features, and then stabilized. Although all 102 features achieved the highest classification accuracy, only the 50 most important features contributed significantly to the classification results. Therefore, considering the computational cost and efficiency, we chose the first 50 most important features to reduce the original number of Sentinel-1A-derived features and used them for the following experiments. 

**Figure 4:**
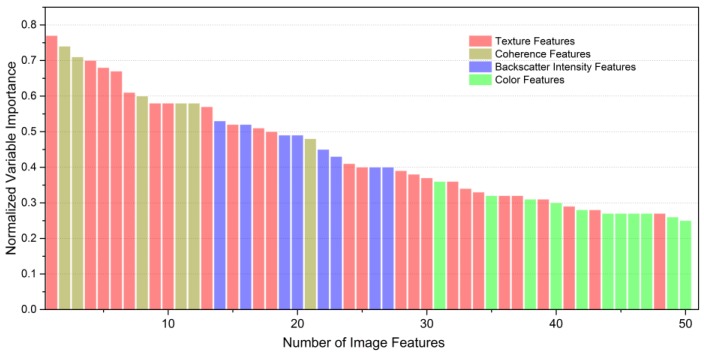
50 first Sentinel-1A-derived features sorted by normalized variable importance.

Figure 4 shows the 50 first Sentinel-1A-derived features ordered by normalized variable importance. According to the feature rankings, the texture features contributed the most to the classification, with the highest value above 0.75, followed by the coherence features and the backscatter intensity features; the color features contributed the least to urban land cover classification. All the coherence features and the backscatter intensity features appeared among the first 50 features, while the color features and texture features did not all appear. In addition, the maximum value of the color feature was about 0.35, which was less than the minimum of the coherence feature and the backscatter intensity feature.

**Figure 5:**
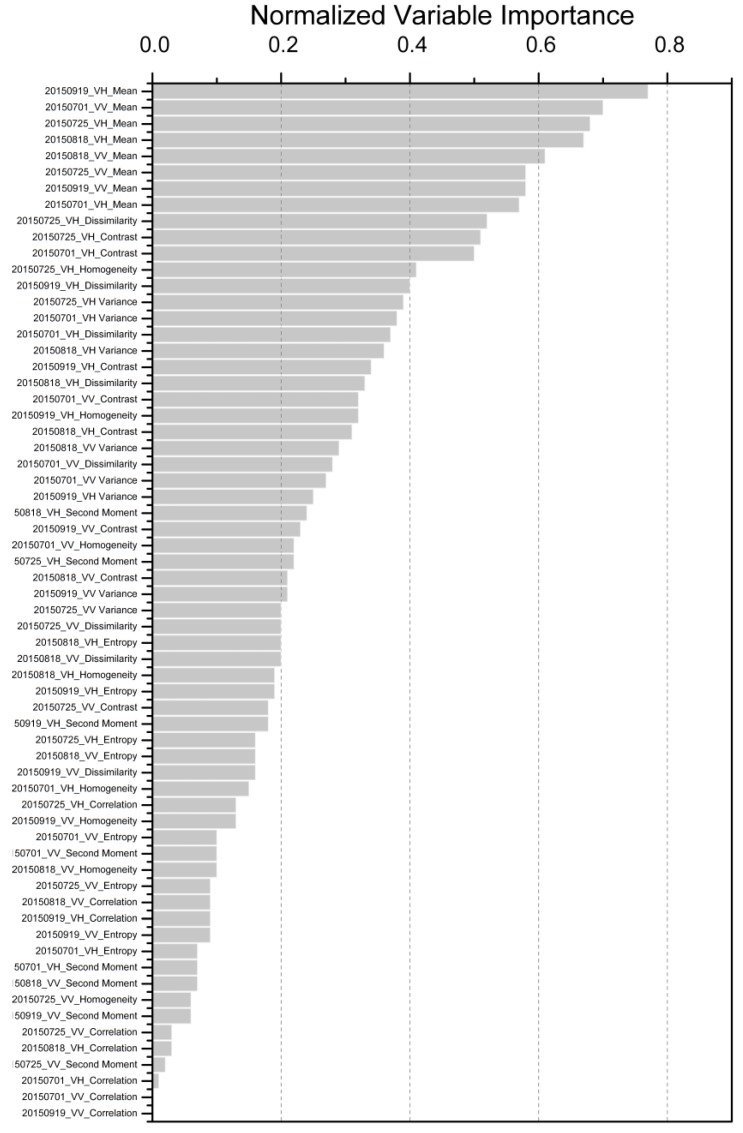
Important contribution variables for different texture features.

Figure 5 presents all the texture features ordered by normalized variable importance. The horizontal axis was labeled according to the polarization of the image (VV or VH), its acquisition date, and the name of the texture feature. By analyzing the individual contributions of the texture features, it is shown that the mean feature contributed the most to the classification, the contributions of which all exceeded 0.5, followed by the dissimilarity, contrast, and variance features; the correlation and angular second moment features contributed the least to urban land cover classification, and their values were all less than 0.25. Finally, there was no significant difference in the contribution of homogeneity and entropy features. Comparing VV and VH polarization information, we found that VH polarization contributed more to the classification than VV polarization; VH polarization appeared 17 times in the first 50 most important features, while VV polarization appeared only 8 times. Jin et al. [87] reported that backscatter coefficients at HV polarization were more important than HH polarization. Compared with co-polarization, cross-polarization has strong sensitivity to vegetation structure and biomass because of its multiple volume scattering [88,89]. In terms of dates, there was no significant difference in the contribution of the texture features of the four Sentinel-1A images, mainly due to the fact that the data came from the same season. In Suzhou, the rainy season is mainly concentrated in the summer and into the plum-rains season in late June. The measure of feature importance provides useful information for selecting the most important classification features, which can reduce the number of inputs to the classification algorithm and thus speed up the classification process [87].

### 4.3. Urban Land-Cover Mapping 

#### 4.3.1. Contribution of Different Feature Combinations

The contribution was assessed by performing RF classifier classification using different feature combinations. These classification results are shown in Table 4. First, SAR images were classified based on four single features, including color features, texture features, coherence features, and backscatter intensity features. As shown in Table 4, the classification accuracy using T was highest, with an overall accuracy of 89.08% (kappa coefficient = 0.8621), followed by VV + VH and C2. Compared with the previous studies [46,90], the classification accuracy of the texture features in this study was higher mainly because each individual texture feature was calculated using their corresponding best window. Although C1 had the worst performance (with an overall accuracy of 58.45% and a kappa coefficient of 0.4734) of the four single features, it could distinguish urban impervious surfaces (BIS and DIS) well. Although the coherence features have a higher contribution to the classification and have good identification ability to the urban impervious surface, their overall classification accuracy is low, mainly due to the low identification ability of the coherence features to forests, grasslands and water bodies. This shows that texture information is more suitable for urban land cover classification and the coherence feature is important information for the extraction of urban impervious surfaces. Some previous studies have shown that crop classification accuracy of backscatter intensity information is higher than that of texture information [26,91]. For all four single features, overall accuracies were lower than 80% except for the value obtained using T. Although the overall accuracy of T was higher than 85%, the corresponding F1 measures of GRA and BIS were lower than 85%, which indicates that four single features could not meet the requirements of urban land cover classification. Consistent with previous studies, classification accuracy of less than 85% when using single polarization image [46] and using only coherence features [92]. The classification accuracy of the backscatter intensity features in this study was higher than previous studies using only single-date dual-polarimetric SAR data [93], mainly due to the use of multi-temporal SAR data. Another previous study using texture information from single-date SAR data obtained producer’s accuracy and user’s accuracy of only 54% and 76% for urban areas, respectively [94]. Therefore, multi-date Sentinel-1A images are needed to obtain better urban land cover identification. For color features and backscatter intensity features, the best classification results were from WAT (with an F1 measure of 84.04% and 91.70%, respectively), followed by FOR; BIS had the worst performance. For texture features, the best performance came from FOR (with an F1 measure of 94.03%), followed by WAT; the poorest performance was from BIS. For coherence features, the beat classification results were found in DIS (with an F1 measure of 73.68%), followed by BIS; GRA had the poorest performance. Therefore, it can be seen that these four different Sentinel-1A-derived features have their own advantages and disadvantages for urban land cover classification. Although texture features had the highest classification accuracy among the four different features, it could not recognize GRA and BIS well. Coherence features were better at identifying urban impervious surfaces, but their overall classification accuracy was lowest. Color and backscatter intensity features were better at recognizing water bodies and forests, but they could not identify BIS well, and their F1 measures were less than 50% for BIS.

To investigate the advantages of the combination of features, classification was carried out by combining two sentinel-1A-derived features. The best classification results were produced using an SAR data combination of T + C1, with an overall accuracy up to 91.55% and a kappa coefficient up to 0.8935, respectively; all F1 measures were above 85%. In general, when the classification accuracy is higher than 85%, the classification result is reliable [43,95]. This shows that SAR images can not only replace optical images for urban land cover classification, but also meet the classification accuracy requirements by using only a combination of texture and coherence features. Furthermore, F1 measures using T + C2 and VV + VH + T were all higher than 85% except BIS. Addition of coherence features derived from SAR images to the backscatter intensity images improved the overall accuracy and kappa coefficient improved by 10.21% and 0.1300, respectively. The importance of coherence features was also supported by the works of Ai et al. [96] and Watanabe et al. [97]. These improvements were also observed when combining two other sentinel-1A-derived features. Uhlmann et al. [98] also showed that the addition of color features can improve the classification accuracy compared with the traditional use of texture features. This indicates that the combined feature performs better than the single feature because the combined feature can provide more useful information for urban land cover classification. For WAT and FOR, the best classification results were from T + C1 (with an F1 measure of 94.93% and 96.00%, respectively), followed by VV + VH + T and T + C2; the poorest classification accuracy came from C1 + C2 and VV + VH + C2. For GRA and BIS, the best accuracy was found in T + C1, followed by T + C2; the worst performance was from VV + VH + C2 and C1 + C2. For DIS, the best F1 measure (89.11%) was with T + C2, followed by T + C1 and VV + VH + T; the poorest performance was from VV + VH + C2, with an F1 measure of 76.77%. This shows that different features extracted from SAR data are suitable for the extraction of different urban land cover types. That is to say, for the extraction of urban land cover, the selection of SAR data features is very important. 

In order to improve classification accuracy and fully explore the ability of Sentinel-1A-derived features to identify urban land cover, a random combination of three features was classified. T + C1 + C2 had the highest classification accuracy (with an overall accuracy of 92.95% and a kappa coefficient of 0.9113), followed by VV + VH + T + C1 (with an overall accuracy of 91.90% and a kappa coefficient of 0.8978); the addition of color features increased overall accuracy and kappa coefficient by 1.4% and 0.0178, respectively; the highest classification accuracy came from FOR (F1 measure = 96.35%), while the lowest classification accuracy came from GRA (F1 measure = 87.72%). VV + VH + C1 + C2 had the worst classification results, with an overall accuracy of 87.85% and a kappa coefficient of 0.8466; unlike the classification results of T + C1 + C2, the best classification results were from WAT (F1 measure = 94.58%) and the worst classification results were from BIS (F1 measure = 76.60%). VV + VH + T + C2 can well identify urban land cover, except for BIS, F1 measures of other land cover types were no less than 85%; adding texture images to the combination of VV + VH + C2 allowed us to increase both the overall accuracy and kappa coefficient from 76.41% to 90.14% and from 0.7009 to 0.8757, respectively. This also indicates that the classification accuracy can be effectively improved when different Sentinel-1A-derived features were combined.

#### 4.3.2. Multi-Sensor Urban Land Cover Mapping

To assess the impact of multi-sensor earth observation data on urban land cover extraction, three different earth observation sensors, including Sentinel-1A, Landsat-8 OLI, and Hyperion, were classified by RF classifiers (see Table 5). First, these earth observation data were classified based on three different single sensors. As shown in Table 5, L had the highest classification accuracy (with an overall accuracy of 95.89% and a kappa coefficient of 0.9480), followed by E. Although the classification accuracy of VV + VH + T + C1 + C2 was the lowest, its classification results were satisfactory, and the classification accuracy of each urban land cover types was higher than 85%; it had the best performance among all combinations of Sentinel-1A-derived features, with an overall accuracy up to 93.13% and a kappa coefficient up to 0.9135, respectively. Compared with previous research results [91], the classification accuracy of SAR data in this study was higher mainly because more SAR-derived features were used. For both Landsat-8 OLI and Hyperion images, the highest classification accuracy came from FOR, with an F1 measure of 98.00% and 97.59%, respectively. The worst classification results for Landsat-8 OLI data came from DIS (F1 measure = 93.52%), while the worst performance for Hyperion data was from GRA (F1 measure = 92.74%). Unlike Landsat-8 OLI and Hyperion, the highest classification accuracy for Sentinel-1A came from WAT (F1 measure = 97.11%); the worst performance came from BIS, with an F1 measure of 89.02%. This shows that the earth observation data of different sensors are suitable for the identification of different land cover types. These sensors record surface information at different wavelengths [99], optical data has more spectral information, and SAR data has richer texture information. 

**Figure 6:**
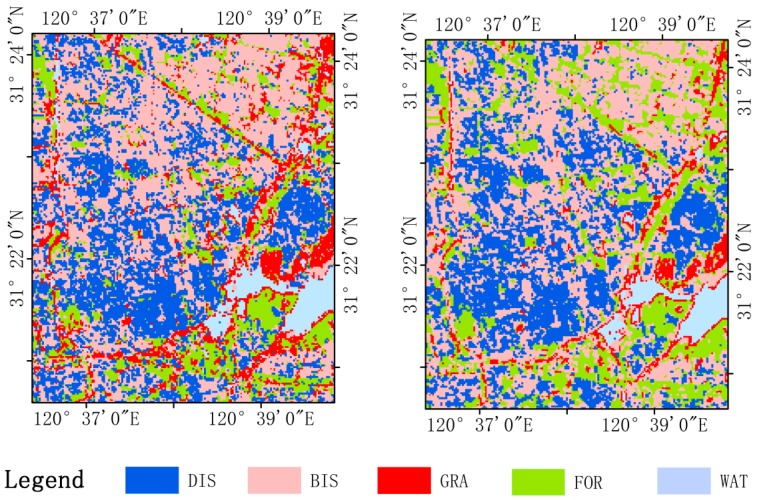
Final map products using the RF classifier. (**Left**) Using the combination of VV + VH + T + C1 + C2 + E. (**Right**) Using the combination of VV + VH + T + C1 + C2.

In order to utilize the multi-source data to potentially improve the classification accuracy, classification was performed by combining three optical and radar instruments. As shown in Table 5, the best classification results were obtained with the combination of Sentinel-1A and Hyperion data, with an overall accuracy up to 99.12% and a kappa coefficient up to 0.9889, respectively; the addition of Sentinel-1A data to Hyperion images increased overall accuracy and kappa coefficient by 4.01% and 0.0519, respectively. These classification results are better than those reported by Kumar et al. [35], in which the overall accuracy of the combination of SAR and hyperspectral data was only 68.90%. The accuracy obtained using the combination of Sentinel-1A and Landsat-8 OLI data was lower, with an overall accuracy of 96.83% and a kappa coefficient of 0.9600; the addition of Sentinel-1A data to Landsat-8 OLI images also resulted in improved classification accuracy. This indicates that the combination of Sentinel-1A and Hyperion data leads to better classification accuracy compared to the combination of Sentinel-1A and Landsat-8 OLI images. The differences in wavelength determine that optical and SAR data will respond to different characteristics of the surface. SAR data contains the structure and dielectric properties of the Earth’s surface material, while optical data provides information on the surface reflectance and emissivity characteristics [100]. Zhang et al. [101] used empirical neural network with Landsat TM and ERS-2 SAR data to improve the estimations of water characteristics and found that microwave data can help improve the estimation of these characteristics. Optical and SAR sensors can use the complementarity of their information to improve accuracy. This explanation of complementary information is also supported by previous literature, which makes use of multispectral and SAR data for land use and land cover identification [30,90,102]. Figure 6 presents samples of the final map products.

## 5. Conclusions

In this study, we evaluated the ability and contribution of using backscatter intensity, texture, coherence, and color features extracted from Sentinel-1A data for urban land cover classification and compared different multi-sensor land cover mapping methods to improve classification accuracy. The following several conclusions can be drawn from these experiments: (1) For the combination of all texture features, the optimal window size was 9 × 9. For each individual texture feature (mean, variance, entropy, dissimilarity, homogeneity, correlation, contrast and angular second moment), the optimal window sizes were: 5 × 5, 23 × 23, 25 × 25, 13 × 13, 19 × 19, 49 × 49, 51 × 51 and 9 × 9, respectively. The mean feature had the best classification result, followed by the dissimilarity; the worst classification result came from the angular second moment and correlation features; (2) The RF importance measures showed that the texture features contribute the most to the classification, with the highest value above 0.75, followed by the coherence features and the backscatter intensity features; the color features contributed the least to urban land cover classification; (3) Among all four different feature types, the classification accuracy using texture features was highest, with an overall accuracy of 89.08% (kappa coefficient = 0.8621), followed by backscatter intensity and color features. Although the coherence features had the worst performance of the four single features, it could distinguish urban impervious surfaces (BIS and DIS) well. Satisfactory classification results can be obtained using only the combination of texture and coherence features, with an overall accuracy up to 91.55% and a kappa coefficient up to 0.8935, respectively; all F1 measures were above 85%. When only Sentinel-1A was utilized, the combination of all Sentinel-1A-derived features performed the best; and (4) Better classification results could be obtained when using multi-sensor for urban land cover mapping. The combination of Sentinel-1A and Hyperion data yielded higher accuracy than the combination of Sentinel-1A and Landsat-8 OLI imagery, with an overall accuracy up to 99.12% and a kappa coefficient up to 0.9889, respectively; the addition of Sentinel-1A data to Hyperion images increased overall accuracy and kappa coefficient by 4.01% and 0.0519, respectively. 

With the further application of Sentinel-1A images, our findings provide a reference guide on how to select and combine multi-type features. In a future study, different SAR-derived features from different seasons will be extracted to investigate the impact of seasonality on urban land cover mapping under different climatic conditions. 

## Figures and Tables

**Table 1:** Acquisition parameters of the Sentinel-1A data acquired in this paper.

Date	Imaging Model	Incident Angle (◦)	Product	Polarization
1 July 2015	IW	39.08	SLC	VV/VH
25 July 2015	IW	39.08	SLC	VV/VH
18 August 2015	IW	39.08	SLC	VV/VH
11 September 2015	IW	39.08	SLC	VV/VH

**Table 2:** Distribution of training and validation data for each class.

Class	Number of Training Pixels	Number of Validation Pixels
WAT	563	577
FOR	544	565
BIS	521	527
DIS	546	531
GRA	506	478

**Table 3:** Different combinations of Sentinel-1A-derived features, Landsat-8 OLI, and Hyperion images.

ID	Combinations Code	Description
1	VV + VH	Backscatter intensity features of all four Sentinel-1A images
2	T	Texture features of all four Sentinel-1A images
3	C1	Coherence features of all four Sentinel-1A images
4	C2	Color features of all four Sentinel-1A images
5	VV + VH + T	Backscatter intensity and texture features of all four Sentinel-1A images
6	VV + VH + C1	Backscatter intensity and coherence features of all four Sentinel-1A images
7	VV + VH + C2	Backscatter intensity and color features of all four Sentinel-1A images
8	T + C1	Texture and coherence features of all four Sentinel-1A images
9	T + C2	Texture and color features of all four Sentinel-1A images
10	C1 + C2	Coherence and color features of all four Sentinel-1A images
11	VV + VH + T + C1	Combination of backscatter intensity, texture, and coherence features of all four Sentinel-1A images
12	VV + VH + T + C2	Combination of backscatter intensity, texture, and color features of all four Sentinel-1A images
13	VV + VH + C1 + C2	Combination of backscatter intensity, coherence, and color features of all four Sentinel-1A images
14	T + C1 + C2	Combination of texture, coherence, and color features of all four Sentinel-1A images
15	L	Landsat-8 data
16	E	EO-1 Hyperion data
17	VV + VH + T + C1 + C2	Combination of backscatter intensity, texture, coherence, and color features of all four Sentinel-1A images
18	VV + VH + T + C1 + C2 + L	Combination of Sentinel-1A (backscatter intensity, texture, coherence, and color features) and Landsat-8 data
19	VV + VH + T + C1 + C2 + E	Combination of Sentinel-1A (backscatter intensity, texture, coherence, and color features) and EO-1 Hyperion data

Notes: VV means VV polarization; VH means VH polarization; T means texture features; C1 means coherence features; C2 means color features; L means Landsat-8 data; E means EO-1 Hyperion data.

**Table 4:** Classification accuracies obtained using different combinations of color features, texture features, coherence features, and backscatter intensity features.

ID	F1 Measure (%)					Overall Accuracy (%)	Kappa
	WAT	FOR	GRA	BIS	DIS		
VV + VH	91.70	80.39	75.74	38.46	76.52	76.23	0.6988
T	93.86	94.03	81.71	79.49	87.76	89.08	0.8621
C1	51.93	51.55	45.94	70.66	73.68	58.45	0.4734
C2	84.40	80.67	59.11	47.57	69.45	71.48	0.6384
VV + VH + T	94.42	94.96	85.21	79.81	87.81	89.44	0.8668
VV + VH + C1	92.75	93.01	85.03	75.00	80.93	86.44	0.8288
VV + VH + C2	92.15	80.91	76.65	38.99	76.77	76.41	0.7009
T + C1	94.93	96.00	87.64	85.71	88.78	91.55	0.8935
T + C2	94.53	94.29	85.72	81.90	89.11	89.96	0.8734
C1 + C2	84.83	82.35	60.44	73.52	81.25	78.17	0.7232
VV + VH + T + C1	94.97	96.40	88.14	86.57	90.38	91.90	0.8978
VV + VH + T + C2	94.93	95.27	85.88	82.14	89.85	90.14	0.8757
VV + VH + C1 + C2	94.58	93.66	86.75	76.60	82.35	87.85	0.8466
T + C1 + C2	95.62	96.35	87.72	88.84	90.73	92.95	0.9113

Notes: The sample code explanations are shown in Table 3 (e.g., VV means VV polarization; VH means VH polarization; T means texture features; C1 means coherence features; C2 means color features).

**Table 5:** Multi-Sensor classification accuracies obtained using different combinations of Sentinel-1A, Landsat-8 OLI, and Hyperion images.

ID	F1 Measure (%)					Overall Accuracy (%)	Kappa
	WAT	FOR	GRA	BIS	DIS		
L	97.52	98.00	94.30	96.04	93.52	95.89	0.9480
E	97.00	97.59	92.74	92.90	93.34	95.11	0.9370
VV + VH + T + C1 + C2	97.11	96.47	90.18	89.02	90.82	93.13	0.9135
VV + VH + T + C1 + C2 + L	97.52	99.28	94.61	95.57	95.61	96.83	0.9600
VV + VH + T + C1 + C2 + E	98.95	99.96	98.16	99.02	99.03	99.12	0.9889

Notes: The sample code explanations are shown in Table 3 (e.g., L means Landsat-8 data; E means EO-1 Hyperion data).

## References

[1] World Health Organization. Centre for Health Development (2010). Hidden Cities: Unmasking and Overcoming Health Inequities in Urban Settings.

[2] Jacobson M.Z., Ten Hoeve J.E. (2012). Effects of urban surfaces and white roofs on global and regional climate. J. Clim..

[3] Shi L.F., Ling F., Ge Y., Foody G.M., Li X.D., Wang L.H., Zhang Y.H., Du Y. (2017). Impervious surface change mapping with an uncertainty-based spatial-temporal consistency model: A case study in wuhan city using landsat time-series datasets from 1987 to 2016. Remote Sens..

[4] Sharma R.C., Tateishi R., Hara K., Gharechelou S., Iizuka K. (2016). Global mapping of urban built-up areas of year 2014 by combining modis multispectral data with viirs nighttime light data. Int. J. Digit. Earth.

[5] Angiuli E., Trianni G. (2014). Urban mapping in landsat images based on normalized difference spectral vector. IEEE Geosci. Remote Sens. Lett..

[6] Ribeiro B.M.G., Fonseca L.M.G. (2013). Urban Land Cover Classification Using Worldview-2 Images and c4.5 Algorithm.

[7] Niu X., Ban Y.F. (2013). Multi-temporal RADARSAT-2 polarimetric SAR data for urban land-cover classification using an object-based support vector machine and a rule-based approach. Int. J. Remote Sens..

[8] Li X., Yeh A.G. (2004). Multitemporal SAR images for monitoring cultivation systems using case-based reasoning. Remote Sens. Environ..

[9] LeeJs P. (2009). Polarimetricradarimaging: From Basics to Applications.

[10] Ulaby F.T., Kouyate F., Brisco B., Williams T.H.L. (1986). Textural information in SAR images. IEEE Trans. Geosci. Remote.

[11] Aschbacher J., Pongsrihadulchai A., Karnchanasutham S., Rodprom C., Paudyal D.R., Toan T.L. Assessment of ers-1 SAR data for rice crop mapping and monitoring. Proceedings of the International Geoscience and Remote Sensing Symposium, IGARSS ‘95. ‘Quantitative Remote Sensing for Science and Applications’.

[12] Park N.W., Chi K.H. (2008). Integration of multitemporal/polarization c-band SAR data sets for land-cover classification. Int. J. Remote Sens..

[13] Dell’Acqua F., Gamba P., Lisini G. (2003). Improvements to urban area characterization using multitemporal and multiangle SAR images. IEEE Trans. Geosci. Remote.

[14] Ban Y.F., Hu H.T., Rangel I., Ju W., Zhao S. (2007). Fusion of radarsat fine-beam SAR and quickbird data for land-cover mapping and change detection. Geoinformatics 2007: Remotely Sensed Data and Information, pts 1 and 2.

[15] Waske B., van der Linden S. (2008). Classifying multilevel imagery from SAR and optical sensors by decision fusion. IEEE Trans. Geosci. Remote.

[16] Mazher A., Li P.J., Weng Q., Gamba P., Xian G., Chen J.M., Liang S. (2016). A decision fusion method for land cover classification using multi-sensor data. Proceedings of the 4th International Workshop on Earth Observation and Remote Sensing Applications.

[17] Bigdeli B., Pahlavani P. (2016). High resolution multisensor fusion of sar, optical and lidar data based on crisp vs. Fuzzy and feature vs. Decision ensemble systems. Int. J. Appl. Earth Obs. Geoinfor..

[18] Xiang D.L., Wang W., Tang T., Su Y. (2017). Multiple-component polarimetric decomposition with new volume scattering models for polsar urban areas. IET Radar Sonar Navig..

[19] Hariharan S., Tirodkar S., Bhattacharya A. (2016). Polarimetric SAR decomposition parameter subset selection and their optimal dynamic range evaluation for urban area classification using random forest. Int. J. Appl. Earth Obs. Geoinfor..

[20] Deng L., Yan Y.N., Sun C. (2015). Use of sub-aperture decomposition for supervised polsar classification in urban area. Remote Sens..

[21] Salehi M., Sahebi M.R., Maghsoudi Y. (2014). Improving the accuracy of urban land cover classification using RADARSAT-2 polsar data. IEEE J. Sel. Top. Appl. Earth Obs. Remote Sens..

[22] Gao L.A., Ban Y.F., Guo H., Wang C. (2010). Multitemporal RADARSAT-2 polarimetric SAR data for urban land-cover mapping. Proceedings of the Sixth International Symposium on Digital Earth.

[23] Balzter H., Cole B., Thiel C., Schmullius C. (2015). Mapping corine land cover from sentinel-1a SAR and srtm digital elevation model data using random forests. Remote Sens..

[24] McNairn H., Kross A., Lapen D., Caves R., Shang J. (2014). Early season monitoring of corn and soybeans with terraSAR-X and radarsat-2. Int. J. Appl. Earth Obs. Geoinfor..

[25] McNairn H., Champagne C., Shang J., Holmstrom D., Reichert G. (2009). Integration of optical and synthetic aperture radar (SAR) imagery for delivering operational annual crop inventories. ISPRS J. Photogr. Remote Sens..

[26] Zhou T., Pan J., Zhang P., Wei S., Han T. (2017). Mapping winter wheat with multi-temporal SAR and optical images in an urban agricultural region. Sensors.

[27] Deng C., Li C., Zhu Z., Lin W., Xi L. (2017). Subpixel urban impervious surface mapping: The impact of input landsat images. ISPRS J. Photogr. Remote Sens..

[28] Xu R., Zhang H.S., Lin H. (2017). Urban impervious surfaces estimation from optical and SAR imagery: A comprehensive comparison. IEEE J. Sel. Top. Appl. Earth Obs. Remote Sens..

[29] Werner A., Storie C.D., Storie J. (2014). Evaluating sar-optical image fusions for urban lulc classification in vancouver Canada. Can. J. Remote Sens..

[30] Shao Z., Fu H., Fu P., Yin L. (2016). Mapping urban impervious surface by fusing optical and SAR data at the decision level. Remote Sens..

[31] Jiang L.M., Liao M.S., Lin H., Yang L.M. (2009). Synergistic use of optical and inSAR data for urban impervious surface mapping: A case study in Hong Kong. Int. J. Remote Sens..

[32] Leinenkugel P., Esch T., Kuenzer C. (2011). Settlement detection and impervious surface estimation in the mekong delta using optical and SAR remote sensing data. Remote Sens. Environ..

[33] Zhou Y., Lin C.X., Wang S.X., Liu W.L., Tian Y. (2016). Estimation of building density with the integrated use of GF-1 PMS and Radarsat-2 data. Remote Sens..

[34] Yang L.M., Jiang L.M., Lin H., Liao M.S. (2009). Quantifying sub-pixel urban impervious surface through fusion of optical and insar imagery. Gisci. Remote Sens..

[35] Kumar V., Agrawal P., Agrawal S. (2017). Alos palsar and hyperion data fusion for land use land cover feature extraction. J. Indian Soc. Remote.

[36] Borghys D., Shimoni M., Degueldre G., Perneel C. Improved object recognition by fusion of hyperspectral and SAR Data. Proceedings 5th EARSeL Workshop on Imaging Spectroscopy.

[37] Shokrollahi M., Ebadi H. (2016). Improving the accuracy of land cover classification using fusion of polarimetric SAR and hyperspectral images. J. Indian Soc. Remote.

[38] Breiman L. (2001). Random forests. Mach. Learn..

[39] Van Beijma S., Comber A., Lamb A. (2014). Random forest classification of salt marsh vegetation habitats using quad-polarimetric airborne SAR, elevation and optical RS data. Remote Sens. Environ..

[40] Furtado L.F.D., Silva T.S.F., Novo E. (2016). Dual-season and full-polarimetric c band SAR assessment for vegetation mapping in the amazon *várzea* wetlands. Remote Sens. Environ..

[41] Corcoran J.M., Knight J.F., Gallant A.L. (2013). Influence of multi-source and multi-temporal remotely sensed and ancillary data on the accuracy of random forest classification of wetlands in Northern Minnesota. Remote Sens..

[42] Mandianpari M., Salehi B., Mohammadimanesh F., Motagh M. (2017). Random forest wetland classification using alos-2 l-band, RADARSAT-2 c-band, and terraSAR-X imagery. ISPRS J. Photogr. Remote Sens..

[43] Skakun S., Kussul N., Shelestov A.Y., Lavreniuk M., Kussul O. (2016). Efficiency assessment of multitemporal c-band RADARSAT-2 intensity and landsat-8 surface reflectance satellite imagery for crop classification in Ukraine. IEEE J. Sel. Top. Appl. Earth Obs. Remote Sens..

[44] Shu Y.M., Li J., Gomes G. (2010). Shoreline extraction from RADARSAT-2 intensity imagery using a narrow band level set segmentation approach. Mar. Geod..

[45] Li Y., Li J. (2010). Oil spill detection from SAR intensity imagery using a marked point process. Remote Sens. Environ..

[46] Mishra V.N., Prasad R., Kumar P., Gupta D.K., Srivastava P.K. (2016). Dual-polarimetric c-band SAR data for land use/land cover classification by incorporating textural information. Environ. Earth Sci..

[47] Zhang H., Lin H., Li Y., Zhang Y., Fang C. (2016). Mapping urban impervious surface with dual-polarimetric SAR data: An improved method. Landsc. Urban Plan..

[48] Uhlmann S., Kiranyaz S. (2014). Classification of dual- and single polarized SAR images by incorporating visual features. ISPRS J. Photogr. Remote Sens..

[49] Pulvirenti L., Chini M., Pierdicca N., Boni G. (2016). Use of SAR data for detecting floodwater in urban and agricultural areas: The role of the interferometric coherence. IEEE Trans. Geosci. Remote.

[50] Xiang D.L., Tang T., Hu C.B., Fan Q.H., Su Y. (2016). Built-up area extraction from polsar imagery with model-based decomposition and polarimetric coherence. Remote Sens..

[51] Zhang X.M., He G.J., Zhang Z.M., Peng Y., Long T.F. (2017). Spectral-spatial multi-feature classification of remote sensing big data based on a random forest classifier for land cover mapping. Clust. Comput..

[52] Inglada J., Vincent A., Arias M., Marais-Sicre C. (2016). Improved early crop type identification by joint use of high temporal resolution SAR and optical image time series. Remote Sens..

[53] Wurm M., Taubenbock H., Weigand M., Schmitt A. (2017). Slum mapping in polarimetric SAR data using spatial features. Remote Sens. Environ..

[54] Schlund M., von Poncet F., Hoekman D.H., Kuntz S., Schmullius C. (2014). Importance of bistatic SAR features from tandem-x for forest mapping and monitoring. Remote Sens. Environ..

[55] Navarro A., Rolim J., Miguel I., Catalão J., Silva J., Painho M., Vekerdy Z. (2016). Crop monitoring based on spot-5 take-5 and sentinel-1a data for the estimation of crop water requirements. Remote Sens..

[56] Zhou L., Guo J., Hu J., Li J., Xu Y., Pan Y., Shi M. (2017). Wuhan surface subsidence analysis in 2015–2016 based on sentinel-1a data by SBAS-inSAR. Remote Sens..

[57] Zhou C., Zheng L. (2017). Mapping radar glacier zones and dry snow line in the antarctic peninsula using sentinel-1 images. Remote Sens..

[58] Ko B., Kim H., Nam J. (2015). Classification of potential water bodies using landsat 8 oli and a combination of two boosted random forest classifiers. Sensors.

[59] Nagne A.D., Dhumal R.K., Vibhute A.D., Rajendra Y.D., Gaikwad S., Kale K.V., Mehrotra S.C. Performance evaluation of urban areas land use classification from hyperspectral data by using mahalanobis classifier. Proceedings of the 2017 11th International Conference on Intelligent Systems and Control (Isco 2017).

[60] Deak M., Telbisz T., Arvai M., Mari L., Horvath F., Kohan B., Szabo O., Kovacs J. (2017). Heterogeneous forest classification by creating mixed vegetation classes using eo-1 hyperion. Int. J. Remote Sens..

[61] Puletti N., Camarretta N., Corona P. (2016). Evaluating eo1-hyperion capability for mapping conifer and broadleaved forests. Eur. J. Remote Sens..

[62] Kar S., Rathore V.S., Champati ray P.K., Sharma R., Swain S.K. (2016). Classification of river water pollution using hyperion data. J. Hydrol..

[63] Chen C., Sui X.X., Zhen G.W., Guo B.Y., Chen X.W. (2016). Extraction of cross-sea bridges from gf-2 pms satellite images using mathematical morphology. Proceedings of the 6th digital earth summit.

[64] Arnold C.L., Gibbons C.J. (1996). Impervious surface coverage: The emergence of a key environmental indicator. J. Am. Plan. Assoc..

[65] Wu C., Murray A.T. (2003). Estimating impervious surface distribution by spectral mixture analysis. Remote Sens. Environ..

[66] Zhang Y., Zhang H.S., Lin H. (2014). Improving the impervious surface estimation with combined use of optical and SAR remote sensing images. Remote Sens. Environ..

[67] Baumann M., Ozdogan M., Kuemmerle T., Wendland K.J., Esipova E., Radeloff V.C. (2012). Using the landsat record to detect forest-cover changes during and after the collapse of the soviet union in the temperate zone of european Russia. Remote Sens. Environ..

[68] Bargiel D. (2017). A new method for crop classification combining time series of radar images and crop phenology information. Remote Sens. Environ..

[69] Schuster C., Schmidt T., Conrad C., Kleinschmit B., Foerster M. (2015). Grassland habitat mapping by intra-annual time series analysis-comparison of rapideye and terraSAR-X satellite data. Int. J. Appl. Earth Obs. Geoinfor..

[70] Arsenault H.H. (1985). Speckle suppression and analysis for synthetic aperture radar images. Opt. Eng..

[71] Zazi L., Boutaleb A., Guettouche M.S. (2017). Identification and mapping of clay minerals in the region of djebel meni (northwestern algeria) using hyperspectral imaging, eo-1 hyperion sensor. Arab. J. Geosci..

[72] Xing Y., Zhang Y., Li N., Wang R., Hu G. (2016). Improved superpixel-based polarimetric synthetic aperture radar image classification integrating color features. J. Appl. Remote Sens..

[73] Manjunath B.S., Ohm J., Vasudevan V.V., Yamada A. (1998). Color and texture descriptors. IEEE Trans. Circuits Syst. Video Technol..

[74] Sun X., Lin X., Shen S., Hu Z. (2017). High-resolution remote sensing data classification over urban areas using random forest ensemble and fully connected conditional random field. ISPRS Int. J. Geo-Inf..

[75] Cheng J., Ji Y., Liu H. (2015). Segmentation-based polsar image classification using visual features: Rhlbp and color features. Remote Sens..

[76] Luo Y., Zhao S., Zhou H., Wang A., He K., Tan L. A novel classification method based on texture analysis using high-resolution SAR and optical data. Proceedings of the International Workshop on Earth Observation & Remote Sensing Applications.

[77] Du P., Samat A., Waske B., Liu S., Li Z. (2015). Random forest and rotation forest for fully polarized SAR image classification using polarimetric and spatial features. ISPRS J. Photogr. Remote Sens..

[78] Rodriguez-Galiano V.F., Chica-Olmo M., Abarca-Hernandez F., Atkinson P.M., Jeganathan C. (2012). Random forest classification of mediterranean land cover using multi-seasonal imagery and multi-seasonal texture. Remote Sens. Environ..

[79] Weydahl D.J. (2001). Analysis of ers SAR coherence images acquired over vegetated areas and urban features. Int. J. Remote Sens..

[80] Hütt C., Koppe W., Miao Y., Bareth G. (2016). Best accuracy land use/land cover (lulc) classification to derive crop types using multitemporal, multisensor, and multi-polarization SAR satellite images. Remote Sens..

[81] Tan W., Liao R., Du Y., Lu J., Li J. Improving urban impervious surface classification by combining landsat and polsar images: A case study in Kitchener-Waterloo, Ontario, Canada. Proceedings of the Geoscience and Remote Sensing Symposium.

[82] Zhang H., Zhang Y., Lin H. Urban land cover mapping using random forest combined with optical and SAR data. Proceedings of the 2012 IEEE International Geoscience and Remote Sensing Symposium (IGARSS).

[83] Rodriguez-Galiano V.F., Ghimire B., Rogan J., Chica-Olmo M., Rigol-Sanchez J.P. (2012). An assessment of the effectiveness of a random forest classifier for land-cover classification. ISPRS J. Photogr. Remote Sens..

[84] Pal M. (2005). Random forest classifier for remote sensing classification. Int. J. Remote Sens..

[85] Pesaresi M. (2000). Texture analysis for urban pattern recognition using fine-resolution panchromatic satellite imagery. Geogr. Environ. Model..

[86] Senthilnath J., Shenoy H.V., Rajendra R., Omkar S.N., Mani V., Diwakar P.G. (2013). Integration of speckle de-noising and image segmentation using synthetic aperture radar image for flood extent extraction. J. Earth Syst. Sci..

[87] Jin H., Mountrakis G., Stehman S.V. (2014). Assessing integration of intensity, polarimetric scattering, interferometric coherence and spatial texture metrics in palsar-derived land cover classification. ISPRS J. Photogr. Remote Sens..

[88] Wolter P.T., Townsend P.A. (2011). Multi-sensor data fusion for estimating forest species composition and abundance in Northern Minnesota. Remote Sens. Environ..

[89] McNairn H., Shang J., Jiao X., Champagne C. (2009). The contribution of alos palsar multipolarization and polarimetric data to crop classification. IEEE Trans. Geosci. Remote.

[90] Zhou T., Zhao M., Sun C., Pan J. (2018). Exploring the impact of seasonality on urban land-cover mapping using multi-season sentinel-1a and gf-1 wfv images in a subtropical monsoon-climate region. ISPRS Int. J. Geo-Inf..

[91] Jia K., Li Q., Tian Y., Wu B., Zhang F., Meng J. (2012). Crop classification using multi-configuration SAR data in the north china plain. Int. J. Remote Sens..

[92] Parihar N.D.A.R.V.S.N.M.S.M.S. (2014). Analysis of l-band SAR backscatter and coherence for delineation of land-use/land-cover. Int. J. Remote Sens..

[93] Silva W.F., Rudorff B.F.T., Formaggio A.R., Paradella W.R., Mura J.C. (2009). Discrimination of agricultural crops in a tropical semi-arid region of brazil based on l-band polarimetric airborne SAR data. ISPRS J. Photogr. Remote Sens..

[94] Roychowdhury K. Comparison between spectral, spatial and polarimetric classification of urban and periurban landcover using temporal sentinel-1 images. Proceedings of the XXIII ISPRS Congress.

[95] Foody G.M. (2002). Status of land cover classification accuracy assessment. Remote Sens. Environ..

[96] Ai L.P., Pang L., Liu H., Sun M.X., He S.G., Bian F., Xie Y. (2016). High resolution SAR coherence and optical fused images applied in land-use cover classification. Geo-Informatics in Resource Management and Sustainable Ecosystem.

[97] Watanabe M., Thapa R.B., Ohsumi T., Fujiwara H., Yonezawa C., Tomii N., Suzuki S. (2016). Detection of damaged urban areas using interferometric SAR coherence change with PALSAR-2. Earth Planets Space.

[98] Uhlmann S., Kiranyaz S. (2014). Integrating color features in polarimetric SAR image classification. IEEE Trans. Geosci. Remote.

[99] Gessner U., Machwitz M., Esch T., Tillack A., Naeimi V., Kuenzer C., Dech S. (2015). Multi-sensor mapping of west african land cover using modis, asar and TanDEM-X/TerraSAR-X data. Remote Sens. Environ..

[100] Zhu Z., Woodcock C.E., Rogan J., Kellndorfer J. (2012). Assessment of spectral, polarimetric, temporal, and spatial dimensions for urban and peri-urban land cover classification using landsat and SAR data. Remote Sens. Environ..

[101] Zhang Y.Z., Pulliainen J., Koponen S., Hallikainen M. (2002). Application of an empirical neural network to surface water quality estimation in the gulf of finland using combined optical data and microwave data. Remote Sens. Environ..

[102] Sameen M.I., Nahhas F.H., Buraihi F.H., Pradhan B., Shariff A.R.B.M. (2016). A refined classification approach by integrating landsat operational land imager (oli) and RADARSAT-2 imagery for land-use and land-cover mapping in a tropical area. Int. J. Remote Sens..

